# Development of a risk score for low back pain in office workers - a cross-sectional study

**DOI:** 10.1186/1471-2474-12-23

**Published:** 2011-01-25

**Authors:** Prawit Janwantanakul, Praneet Pensri, Patriya Moolkay, Wiroj Jiamjarasrangsi

**Affiliations:** 1 Department of Physical Therapy, Faculty of Allied Health Sciences, Chulalongkorn University, Bangkok, 10330, Thailand; 2 Department of Preventive and Social Medicine, Faculty of Medicine, Chulalongkorn University, Bangkok, 10330, Thailand

## Abstract

**Background:**

Low back pain (LBP) is common among office workers and is the most common cause of work-related disability in people under 45 years of age. The aetiology of LBP is widely accepted to be multi-factorial. Prognostic research into office workers at risk of developing LBP has received limited attention. The aims of this study were to develop a risk score to identify office workers likely to have LBP and to evaluate its predictive power.

**Methods:**

397 office workers filled out a self-administered questionnaire and underwent physical examination. The questionnaire gathered data on individual, work-related physical and psychosocial data as well as the presence of low back pain in the previous 4 weeks. The physical examination included measurement of body weight, height, waist circumference, hamstrings length, spinal scoliosis, spinal curve, Backache Index and lumbar stability. Logistic regression was used to select significant factors associated with LBP to build a risk score. The coefficients from the logistic regression model were transformed into the components of a risk score.

**Results:**

The model included six items: previous history of working as an office worker, years of work experience, continuous standing for >2 hrs/d, frequency of forward bending during work day, chair having lumbar support and Backache Index outcome. The risk score for LBP in office workers (The Back pain Risk score for Office Workers: The BROW) was built with a risk score ranging from 0 to 9. A cut-off score of ≥4 had a sensitivity of 80% and a specificity of 58%. The positive predictive value and negative predictive values were 70% each.

**Conclusions:**

The BROW is easy and quick to administer. It appears to have reasonable sensitivity, specificity, positive predictive value and negative predictive values for the cut-off point of ≥4. The BROW is a promising tool for use to identify office workers in need of early interventions. Further prospective study is needed to validate the predictive performance of the BROW.

## Background

Low back pain (LBP) is a major health problem with two thirds of adults suffering from LBP at some time in their lives [1] and approximately 12% to 44% have LBP at any given time [2]. LBP is common among office workers with the one-year prevalence ranging from 23% to 38% [3-5]. LBP is the most common cause of work-related disability in people under 45 years of age and the most expensive cause of work-related disability, in terms of workers' compensation and medical expenses [6]. In the USA, the total yearly cost of LBP exceeds 100 billion US dollars [7].

The etiology of LBP is not fully understood but is assumed to be of multi-factorial origin, indicating that individual, physical and psychosocial factors can contribute to their development and persistence [8]. Previous studies have identified several individual factors associated with LBP including female [4], level of education [9], smoking [10], sleep deprivation [11], prolonged driving [12]. Regarding work-related risk factors, accumulated computer usage has been linked to increased risk of LBP [13]. Sitting for more than half a work day in combination with awkward postures or frequently working in a forward bent position has been found to increase the likelihood of having LBP [14,15]. Poor workstation ergonomics has been shown to significantly contribute to the development of LBP [15]. Various psychosocial problems, such as high stress [16], low job satisfaction [15], low social support [17] and effort-reward imbalance [18] also contributed to an increased occurrence of LBP. Clinical factors, such as scoliosis [19], low back muscle endurance [20], poor lumbar stability [21] and abnormal trunk mobility [22,23], has been linked to increased risk of LBP.

Having a screening tool for LBP is necessary for several reasons. First, a screening tool provides information about individuals' risk of developing LBP, which will guide health professionals and individuals in joint decisions on further intervention. Identification of persons at risk would also mean the enhancement of resource allocation to those most in need and most likely to benefit from it. Without a screening tool, a large number of people would receive intervention, which is likely to compromise its effectiveness [24,25]. Second, a screening tool allows an examination to be held in primary health care and workplace settings where full clinical examinations are impractical due to limited personnel and time [26]. Lastly, a screening tool is beneficial for selecting relevant individuals for therapeutic research [25].

In a primary care setting, Hill et al [27] developed a brief screening tool for identifying subgroups of patients to guide the provision of early secondary prevention in primary care. The screening tool included 9 items: referred leg pain, co-morbid pain, disability (2 items), bothersomeness, catastrophizing, fear, anxiety and depression. Von Korff and Miglioretti [28] developed a risk score to identify those at risk of chronic LBP. The items in the risk score included pain severity variables (average, worst and current pain intensity), interference with usual activities, work/housework activities and family/social activities, number of other pain and number of days with back pain in the prior six months. To our knowledge, no screening tool to identify office workers at risk for developing LBP has been established. Therefore, the specific purpose of the present study was to develop a risk score to assist health care providers in identifying office workers who were at risk of developing LBP. The aim was achieved by identifying important biopsychosocial predictors, assigning relative weights to each predictor and then estimating the model's predictive performance.

## Methods

A cross-sectional study was conducted in Chulalongkorn University. An invitation letter and information about the study were sent to office workers in 21 departments within the university. Office workers were included if aged between 18-60 years and their job involved office work with at least one year of experience in the current position. They were excluded if they reported pregnancy or spinal, intra-abdominal or femoral surgery in the past year, a history of trauma or accidents at the low back region or had been diagnosed with rheumatoid arthritis, ankylosing spondylitis, systemic lupus erythymatosus or osteoporosis. Those who had any contraindications for physical tests, such as cardiovascular diseases or severe pulmonary diseases, were also excluded. A written informed consent was obtained from all participants. The study was approved by the University Human Ethics Committee.

Subjects filled out a self-administered questionnaire and underwent physical examination, which took approximately 45 minutes to complete. The questionnaire gathered data on individual, work-related physical and psychosocial data as well as the presence of low back pain.

*Individual factors *included age, gender, educational level, marital status, leisure activities, frequency of weekly exercise sessions, quality of sleep, average number of sleeping hours a night, smoking habits and average number of driving hours a day.

Respondents were also asked to complete the physical component of the Thai abbreviated version of World Health Organization Quality of Life (WHOQOL-BREF-Thai), which consists of seven items assessing self-perception of physical health status. Items are scored on a 5-point Likert scale with responses ranging from 1 (not at all) to 5 (an extreme value/very satisfied). The total score ranges from 7 to 35 and are categorized into poor (score of 7-16), fair (17-26) and good quality of life (27-35) [29].

*Work-related physical factors *included the average number of working hours a day as well as working day a week, years of working experience and previous history of working as office workers. Respondents were asked about whether they continuously sat or stood for >2 hours a day and the frequency of using a computer, performing various activities during work (i.e. reaching, forward bending, twisting, lifting moderate to heavy objects and walking) and rest breaks. The questionnaire also asked respondents to self-rate the ergonomics of their workstations (i.e. height of desk and chair, the presence of lumbar support as well as the position of computer screen, keyboard and mouse) and work environment conditions (i.e. temperature, light intensity, noise level, air circulation).

*Psychosocial factors *included the Suanprung stress test (SPST-20) [30,31] and Thai version of Effort-Reward Imbalance questionnaire (Thai ERIQ) [32].

1. The Suanprung stress test consists of 20 items assessing stress level. Items are scored on a 5-point Likert scale with item responses ranging from 0 (no stress) to 5 (extremely high stress). The total score of the test ranges from 0 to 100. Total scores are categorized into 4 levels: low stress (0-24), medium stress (25-42), high stress (43-62) and extremely high stress (63-100) [30,31]

2. The Thai ERIQ consists of 23 items containing 3 subscales: 6 items on effort (the demands and obligations put on the working person), 11 items on reward (offered or promised as part of social exchange in terms of money, esteem and job security/career opportunity) and 6 items on over-commitment (personal coping with demands and reward expectancies). Responses to the items of effort and reward are scored on a 5-point Likert scale (disagree/agree and 4 levels of being distressed) and items of over-commitment are scored on a 4-point Likert scale (strongly disagree, disagree, agree, and strongly agree). Total scores range from 6 to 30 for effort, 11 to 55 for reward and 4 to 14 for over-commitment. A ratio between two scales of effort and reward (weighted by the item number) is calculated to assess the degree of imbalance between high effort expended and low reward received. Effort-reward ratio >1 indicates high level of stressful experience at work. A high score of over-commitment indicates high level of over-commitment [32].

*Low back pain *A picture of the body from the standardized Nordic questionnaire [33] and the question "Have you had low back pain in the previous four weeks?" were included in the questionnaire.

Each participant underwent a physical examination conducted by trained physical therapists according to standardized protocol. The physical examination included the following:

1. Body weight and height were measured by electronic digital scale and a wall-mounted standiometer, respectively.

2. Waist circumference was measured midway between the lower rib margin and the superior border of the iliac crest using a tape measure [34,35].

3. Hamstring length assessment used a passive straight-leg-raising test. With the subject in the supine position, an examiner passively flexed a subject's hip with the knee extended until a subject felt strong stretching in the posterior aspect of the leg. The examiner then recorded the hip angle [36]. A hip angle of ≥80 degree indicates normal hamstring length [37].

4. Spinal scoliosis. The subject was asked to flex forward and the examiner observed the spine from the "skyline" view. The examiner looked for a hump on one side and a hollow on the other, indicating spinal scoliosis [38].

5. Spinal curve measurement using a flexicurve. While the subject stood relaxed, a flexicurve was pressed against the subject's back so that the upper end of the flexicurve was set at the C7 spinous process and the lower end placed at the lumbosacral joint level. The spinal curve from the flexicurve was then traced on a paper and the indexes of thoracic and lumbar curvature were calculated according to Milne and Lauder [39].

6. Backache Index (BAI). The test consists of five active motions of trunk in a standing position. The examiner made her assessment by means of a scoring system that includes pain factors obtained by asking the subject and combined with the stiffness estimation at the end of different lumbar motions. Each movement was scored on the 4-point scale. The sum of the five outcomes yields a BAI with a maximum of 15 points. The higher the score indicates more restrictive spinal movement [40].

7. Lumbar stability test. The test is used to assess the isometric contraction of abdominal and back muscles, which provide lumbar stability. A pressure sensor (Chattanooga, USA.) was placed between L1 and S2 with the subject in the supine position to detect motion. A series of 7 exercises, which required increasing levels of muscular control of the lumbar spine for stability, was performed by each subject. The subject received a pass or fail for each exercise level based on the pressure gauge readings and the absence of movement compensations. The examiner recorded the highest exercise level that the subject attained [41].

Before data collection, the repeatability of data from the questionnaire and physical examination outcomes was assessed on 30 office workers. Each subject was tested twice on two separate days with a week lapse between the measurements.

### Statistical analyses

For the reliability study, the intraclass correlation coefficient (ICC [1,1]), Kappa coefficient and Kappa coefficient with linear weighting were calculated for continuous, nominal and ordinal data, respectively.

Characteristics of subjects participating in the main study were described using means or proportions. To develop a risk score, a series of statistical analyses were conducted. The associations between each factor and LBP in the previous four weeks were evaluated using the bivariate logistic regression analyses. Any factors with a p-value ≤ 0.1 were eligible for addition into multivariate analysis. To retain the statistical power of the database, missing data were handled by using the 'hot-deck imputation' procedure. A respondent was selected at random from the total sample of the study and the value for that person was assigned to a case in which this information was missing. This procedure was conducted repeatedly for each missing value until the dataset was complete [42]. Multiple logistic regression analysis with backward stepwise selection was then performed to determine the optimal combination of biopsychosocial factors needed to predict LBP. Statistical significance was set at the 5% level.

A simplified scoring system was devised on the basis of multiple logistic regression analytical results. Score was assigned to each variable based on the magnitude of the β coefficient. A total score for the risk of developing LBP was calculated as the sum of each variable. A receiver-operating characteristic curve (ROC) and the area under the ROC (AUC) were produced to evaluate the discriminatory ability of the risk score. Sensitivity, specificity, positive predictive value (PPV) and negative predictive value (NPV) for several cut-off scores were calculated. The cut-off score that gave the maximum sum of sensitivity and specificity was taken as an optimum. All statistical analyses were performed using SPSS statistical software, version 17.0 (SPSS Inc, Chicago, IL, USA).

## Results

The results of reliability study showed moderate (0.49) to very good (1.00) repeatability of the outcomes [43]. A total of 454 office workers participated in the study. Of these, 51 were excluded because they did not meet the inclusion criteria and 6 were excluded because they did not provide answers about their LBP in the previous four weeks. Thus, the final analysis was based on the data collected from 397 office workers.

Most respondents were females with Bachelor's degree. Their working time resembled typical office workers (i.e. 8 hours per day and 5 days per week). The average body mass index of this sample was slightly above normal range for Asians [44]. Nearly all office workers reported fair to good quality of life but about half of them experienced high to extremely high stress (Table 1).

**Table 1:** Characteristics of study population

Characteristics	N (%)	Mean ± SD
**Gender **(n = 397)		
Male	65 (16.4)	
Female	332 (83.6)	
**Age(years) **(n = 396)		41.5 ± 9.7
**Body mass index (Kg/m^2^) **(n = 395)		23.9 ± 4.6
**Level of education **(n = 386)		
Primary school	11 (2.8)	
Secondary school	16 (4.1)	
College	41 (10.6)	
Bachelor's degree	209 (54.1)	
Higher than Bachelor's degree	109 (28.2)	
**Years of work experience (years) **(n = 362)		14.9 ± 9.0
**Weekly working days (days per week) **(n = 363)		5.1 ± 0.6
**Daily working hours (hours per day) **(n = 372)		8.0 ± 1.3
**Quality of life measured by the WHOQOL-BREF-Thai **(n = 391)		25.7 ± 3.5
Poor	4 (1.0)	
Fair	225 (57.5)	
Good	162 (41.4)	
**Stress level measured by the Suanprung stress test **(n = 391)		42.8 ± 17.3
Low	62 (15.9)	
Medium	131 (33.5)	
High	149 (38.1)	
Extremely high	49 (12.5)	

Two hundred and nineteen participants (55%) reported LBP during the previous four weeks. A varying number of office workers reported frequent computer use (92%), sitting for >2 hours a day (88%), forward bending (58%), lifting moderate to heavy objects (52%), body twisting (46%), reaching (38%) and standing for >2 hours a day (22%) during the work day. A majority of participants reported satisfaction with the positions of the computer screen (68%) and keyboard/mouse (55%). Forty-five per cent of participants used a chair with lumbar support.

When performing bivariate logistic regression analyses, factors showing p-value ≤ 0.1 were body weight, waist circumference, sleep quality, smoking habits, previous history of working as an office worker, years of work experience, continuous standing for >2 hrs a day, frequency of computer use, reaching, forward bending, body twisting, lifting moderate to heavy objects and rest breaks, self-perception of desk height, chair height and keyboard/mouse positions, chair having lumbar support, the WHOQOL-BREF-Thai score, the Suanprung stress test score, lumbar stability test outcome, Backache Index outcome and Effort-Reward ratio. There was a significant correlation between body weight and waist circumference. Thus, body weight was selected for further analysis.

When performing multivariable logistic regression analyses, the results revealed that previous history of working as office workers, years of work experience, continuous standing for >2 hrs a day, frequency of forward bending, chair having lumbar support, Backache Index outcome and Effort-Reward ratio strongly correlated with complaints of LBP in the preceding four weeks (Table 2). To develop a risk score for low back pain in office workers, scores were then assigned to each variable, which resulted in a range from 0 to 14 (Table 3). The optimal cut-off score was ≥5 (sensitivity = 74%; specificity = 63%) (Table 4). The PPV and NPV for the cutoff score of ≥5 were 72% and 66%. The area under curve (AUC) was 0.73 (95%CI 0.68-0.78).

**Table 2:** Prevalence and adjusted odds ratio (OR_adj_) with 95% confidence interval (95%CI) of low back pain with respect to factors in the final modelling (n = 397)

Factors	N	Prevalence n (%)	OR_adj_	95% CI	P
**Previous history of working as an office worker**					
Yes	173	109 (63.0)	1.72	1.08-2.74	0.023
No	224	110 (49.1)	1.00		
**Years of work experience (years)**					
< 10	122	54 (44.3)	1.00		
10-19	146	96 (65.8)	3.48	1.95-6.20	< 0.001
≥20	129	69 (53.5)	2.03	1.14-3.62	0.017
**Continuous standing >2 hrs/d**					
Yes	86	55 (64.0)	1.86	1.03-3.33	0.039
No	311	164 (52.7)	1.00		
**Frequently bending forward during the work day**					
Yes	228	137 (60.1)	1.78	1.12-2.83	0.014
No	169	82 (48.5)	1.00		
**Chair having lumbar support**					
Yes	177	88 (49.7)	1.00		
No	220	131 (59.5)	1.65	1.04-2.63	0.035
**Backache Index outcome**					
0	197	79 (40.1)	1.00		
≥1	200	140 (70.0)	4.02	2.52-6.42	< 0.001
**Effort-Reward ratio**					
≤ 1	382	206 (53.9)	1.00		
>1	15	13 (86.7)	6.41	1.25-32.87	0.026

**Table 3:** Risk scores for low back pain with the inclusion of the Thai ERIQ

Factors	Beta Coefficient	Risk score*
**Previous history of working as an office worker**		
Yes	0.54	1
No		0
**Years of work experience (years)**		
< 10		0
10-19	1.25	3
≥20	0.71	1
**Continuous standing >2 hrs/d**		
Yes	0.62	1
No		0
**Frequently bending forward during the work day**		
Yes	0.58	1
No		0
**Chair having lumbar support**		
Yes		0
No	0.50	1
**Backache Index**		
0		0
≥1	1.39	3
**Effort-Reward ratio**		
≤ 1		0
>1	1.86	4

* Reference groups were assigned a score of 0. β Coefficient of chair without lumbar support was assigned a score of 1 and then the other β Coefficient was multiplied by 2 (1/0.50) and rounded off to the nearest integer.

**Table 4:** Sensitivity and specificity of each cut-off value for the risk score for low back pain with the inclusion of the Thai ERIQ

Cut-off value	Sensitivity	Specificity	PPV	NPV
≥ 1	100.0	2.1	56.2	100.0
≥ 2	98.4	11.7	58.3	85.0
≥ 3	90.7	28.3	61.3	70.7
≥ 4	83.5	48.3	60.0	70.0
≥ 5	74.2	63.4	71.8	66.2
≥ 6	51.6	78.6	75.2	56.4
≥ 7	30.2	91.0	80.9	51.0
≥ 8	22.5	95.2	85.4	49.5
≥ 9	8.2	97.9	83.3	46.0
≥ 10	3.3	100.0	100.0	45.2
≥ 11	2.2	100.0	100.0	44.9

Because the Thai ERIQ consists of 23 questions, the model of the risk score with the exclusion of the Thai ERIQ was examined for its predictive power. The score ranged from 0 to 9 (Table 5). The optimal cut-off score was ≥4 for a sensitivity of 80% and a specificity of 58% (Table 6). The PPV and NPV for the cut-off score of ≥4 were 70% each. The predictive ability was unchanged as compared with the model that included the Thai ERIQ (AUC 0.73, 95%CI 0.68-0.78).

**Table 5:** Risk scores for low back pain with the exclusion of the Thai ERIQ

Factors	Beta Coefficient	Risk score*
**Previous history of working as an office worker**		
Yes	0.51	1
No		0
**Years of work experience (years)**		
< 10		0
10-19	1.17	2
≥20	0.62	1
**Continuous standing >2 hrs/d**		
Yes	0.59	1
No		0
**Frequently bending forward during the work day**		
Yes	0.55	1
No		0
**Chair having lumbar support**		
Yes		0
No	0.48	1
**Backache Index**		
0		0
≥1	1.41	3

* Reference groups were assigned a score of 0. β Coefficient of chair without lumbar support was assigned a score of 1 and then the other β Coefficient was multiplied by 2 (1/0.50) and rounded off to the nearest integer.

**Table 6:** Sensitivity and specificity of each cut-off value for the risk score for low back pain with the exclusion of the Thai ERIQ

Cut-off value	Sensitivity	Specificity	PPV	NPV
≥ 1	100.0	1.9	56.0	100.0
≥ 2	98.4	12.3	58.3	86.4
≥ 3	88.5	33.1	62.3	69.9
≥ 4	79.7	57.8	70.2	69.5
≥ 5	63.0	69.5	72.0	60.1
≥ 6	44.3	83.8	77.3	54.7
≥ 7	23.4	95.5	86.5	50.0
≥ 8	6.8	98.1	81.2	45.8
≥ 9	0.5	100.0	100.0	44.6

## Discussion

The current study aimed to build a screening tool to justify office workers at risk of developing LBP. Various individual, work-related physical and psychosocial factors as well as outcomes from physical examination conducted by trained physical therapists were included in the analysis. Data were collected from office workers in workplace settings. The results showed that a risk score for LBP in office workers or "The Back pain Risk score for Office Workers (The BROW)" comprised the following six items to calculate the total score: previous history of working as an office worker, years of work experience, continuous standing for >2 hrs a day, frequency of forward bending during the work day, chair having lumbar support, BAI outcome. Each item is unequal in weight. The score ranged from 0 to 9 and the higher the score indicates higher risk of LBP.

The Thai ERIQ was excluded from the final version of the BROW because of three reasons. First, the inclusion of the Thai ERIQ would make the BROW cumbersome and time consuming. Second, the exclusion of the Thai ERIQ from the BROW did not alter the predictive ability, as measured by the AUC, of the risk score and the AUC is still at an acceptable level. This is because in this study the number of office workers, who had effort-reward imbalance, was small (14/370). Third, the exclusion of the Thai ERIQ would make the BROW accessible for those for whom the Thai ERIQ is unavailable.

The strongest predictor in the BROW was the BAI outcome (OR_adj _= 4.02). The BAI is a tool aimed at assessing overall restricted spinal movement in cases of LBP [40]. Since computer use and document work require a sitting posture, the strong association between the BAI outcome and the risk of LBP may relate to the prolonged sitting posture causing lumbar stiffness. Evidence suggests that prolonged sitting may lead to lumber stiffness, which may predispose the lumbar spine to injury during forceful loading [45].

The BROW is relatively easy to administer and can be carried out within a short space of time because it mainly relies on subjective information from an individual. However, the disadvantage of the BROW is the requirement to perform the BAI test with a health professional. Although the BAI test is quite easy to perform, health professionals with less experience in doing the test may misclassify the outcome, which potentially compromises the risk score's predictive performance. Within this limitation, the BROW is a promising tool for the early identification of office workers at risk of developing LBP, who will receive the greatest benefit from preventive intervention. The BROW is suitable for utilising in primary health care and workplace settings where a full clinical examination is impractical due to limited personnel and time.

Selection of an optimal cut-off point largely depends on the purpose of utilizing the risk score and requires knowledge of the sensitivity and specificity of a risk score. With a cut-off score of ≥4, the sensitivity, which represents the ability of the risk score to recognize high-risk office workers when present, is 80% and the specificity, which indicates the ability of the risk score to recognize low-risk office workers when present, is 58%. Subsequently, the false positive rate was 42%, meaning that 42% of low-risk office workers will be identified as positive. Because these low-risk workers are unlikely to benefit from a preventive intervention, a high false positive rate would increase intervention cost and time loss. On the other hand, the false negative rate for the cut-off point of ≥4 was 20%, indicating that 20% of high-risk workers will be missed. Because these high-risk workers may not have received deserved preventive intervention, a high false negative rate would cause greater medical expenses and disability from a disease later on. Although the fundamental purpose of having a screening tool is to reduce the number of office workers with LBP, one needs to consider the expected consequences of missing a person at risk as opposed to including a person in an intervention even though they are not at risk. Since LBP is a non-fatal disease and taking into account the circumstances of limited resources one may want to increase the likelihood of including those who are truly at risk of developing LBP. Thus, a screening tool with high specificity would be preferable to high sensitivity. With increasing the cut-off point to the score of ≥6, the specificity increases to 84%, while the sensitivity drops to 44%. The false positive rate decreased to 16%, meaning that less low-risk workers will be identified as positive. Consequently, money and time could be saved.

In practice, predictive values may be more useful for applying the risk score in clinical decision making than sensitivity and specificity rates because predictive values indicate the probability that the result is correct [46]. The results show that the predictive value of the cut-off point of ≥4 was reasonably high. The PPV was 70%, indicating that 70% of subjects with a score of ≥4 are actually at risk of developing LBP. The NPV was 70%, meaning that 70% of subjects with a score of < 4 are not at risk of developing LBP. Although the PPV and NPV provide useful information for interpreting the results of the risk score, they are highly dependent on the prevalence of the condition of interest in the sample [46]. The lower the prevalence of LBP in a population, the more likely a positive test will be a false positive [47]. In this study, the prevalence of 4-week LBP is considered to be high compared to that reported in previous epidemiological studies [48]. The use of a small convenience sample of office workers may lead to selection bias and, consequently, restricts the external validity of this study. Thus, caution must be exercised when using the BROW in other populations.

There are two methodological limitations that are noteworthy. First, the cross-sectional nature of the study only allows the association between exposure and outcome to be examined. It is not possible to establish the causal relation between exposure and outcome. For example, it is unclear if restricted spinal movement, as assessed by the BAI, causes LBP or LBP leads to restricted spinal movement. Therefore, the findings of the present study should be taken as a preliminary result. A prospective study is needed to validate the finding of this study. Second, predictive performance of the BROW was tested on the same population in which the risk score was developed. The model is likely to perform better in the development sample than in an independent sample. In other words, the predictive power is likely to be inflated [24,25]. In addition, the risk score may be very specific to the population studies. Thus, extrapolation of these results to other populations should be made with caution. Further validation and impact studies of the BROW in a new population of office workers are suggested.

## Conclusions

The risk score for LBP in office workers (The BROW) was developed and it contained 6 items with scores ranging from 0 to 9. Using a cut-off score of ≥4, the sensitivity was found to be 80% and the specificity 58%. The positive predictive value and negative predictive value were both 70%. The BROW is easy and quick to complete by health care providers. The BROW is a potentially useful tool in helping clinicians make decisions about office workers' risk of developing LBP. However, further research is required to validate the BROW.

## Competing interests

The authors declare that they have no competing interests.

## Authors' contributions

The authors have contributed in the following ways: PJ and PP provided concept/research design, data analysis and manuscript writing. PM provided data collection, data analysis and manuscript writing. WJ provided concept/research design and manuscript writing. All authors read and approved the final manuscript.

## Pre-publication history

The pre-publication history for this paper can be accessed here:

http://www.biomedcentral.com/1471-2474/12/23/prepub

## References

[B1] Andersson GB Frymoyer JW The epidemiology of spinal disorders The Adult Spine: Principles and Practice 1997 2 New York: Raven Press 93 141

[B2] Koes B Van Tulder M Acute low back pain Am Fam Physician 2006 74 803 5 16970025

[B3] Janwantanakul P Pensri P Jiamjarasrangsri W Sinsongsook T Prevalence of self-reported musculoskeletal symptoms among office workers Occup Med (Lond) 2008 58 436 8 10.1093/occmed/kqn072 18544589

[B4] Juul-Kristensen B Sogaard K Stroyer J Jensen C Computer users' risk factors for developing shoulder, elbow and back symptoms Scand J Work Environ Health 2004 30 390 8 15529802 10.5271/sjweh.827

[B5] Omokhodion FO Sanya AO Risk factors for low back pain among office workers in Ibadan, Southwest Nigeria Occup Med (Lond) 2003 53 287 9 10.1093/occmed/kqg063 12815127

[B6] Andersson GBJ Epidemiologic features of chronic low-back pain Lancet 1999 354 581 5 10.1016/S0140-6736(99)01312-4 10470716

[B7] Katz JN Lumbar disc disorders and low-back pain: socioeconomic factors and consequences J Bone Joint Surg Am 2006 88 21 4 10.2106/JBJS.E.01273 16595438

[B8] Manchikanti L Epidemiology of low back pain Pain Physician 2000 3 167 92 16906196

[B9] Dionne CE von Korff M Koepsell TD Deyo RA Barlow WE Checkoway H Formal education and back pain: a review J Epidmiol Community Health 2001 55 455 68 10.1136/jech.55.7.455 PMC1731944 11413174

[B10] Shiri R Karppinen J Leino-Arjas P Solovieva S Viikari-Juntura E The association between smoking and low back pain: a meta-analysis Am J Med 2010 123 87.e7 87.e35 10.1016/j.amjmed.2009.05.028 20102998

[B11] Muto S Muto T Seo A Yoshida T Taoda K Watanabe M Prevalence of and risk factors for low back pain among staffs in schools for physically and mentally handicapped children Ind Health 2006 44 113 7 10.2486/indhealth.44.123 16610547

[B12] Chen JC Chang WR Chang W Christiani D Occupational factors associated with low back pain in urban taxi drivers Occup Med (Lond) 2005 55 535 40 10.1093/occmed/kqi125 16141293

[B13] Ortiz-Hernández L Tamez-González S Martínez-Alcántara S Méndez-Ramírez I Computer use increases the risk of musculoskeletal disorders among newspaper office workers Arch Med Res 2003 34 331 42 12957532 10.1016/S0188-4409(03)00053-5

[B14] Lis AM Black KM Korn H Nordin M Association between sitting and occupational LBP Eur Spine J 2007 16 283 98 10.1007/s00586-006-0143-7 16736200 PMC2200681

[B15] Spyropoulos P Papathanasiou G Georgoudis G Chronopoulos E Koutis H Koumoutsou F Prevalence of low back pain in Greek public office workers Pain Physician 2007 10 651 9 17876361

[B16] Yip YB Ho SC Chan SG Socio-psychological stressors as risk factors for low back pain in Chinese middle-aged women J Adv Nurs 2001 36 409 16 10.1046/j.1365-2648.2001.01988.x 11686755

[B17] Clays E De Bacquer D Leynen F Kornitzer M Kittel F De Backer G The impact of psychosocial factors on low back pain: longitudinal results from the Belstress study Spine 2007 32 262 8 10.1097/01.brs.0000251884.94821.c0 17224824

[B18] Rugulies R Krause N Effort-reward imbalance and incidence of low back and neck injuries in San Francisco transit operators Occup Environ Med 2008 65 525 33 10.1136/oem.2007.035188 18056748

[B19] Gremeaux V Casillas JM Fabbro-Peray P Pelissier J Herisson C Perennou D Analysis of low back pain in adults with scoliosis Spine 2008 33 402 5 10.1097/BRS.0b013e318163fa42 18277872

[B20] Hamberg-van Reenen HH Ariens GA Blatter BM Twisk JW van Mechelen W Bongers PM Physical capacity in relation to low back, neck, or shoulder pain in a working population Occup Environ Med 2006 63 371 7 10.1136/oem.2006.026914 16709701 PMC2078108

[B21] Hodges PW Richardson CA Inefficient muscular stabilization of the lumbar spine associated with low back pain. A motor control evaluation of transversus abdominis Spine 1996 21 2640 50 10.1097/00007632-199611150-00014 8961451

[B22] Adams MA Mannion AF Dolan P Personal risk factors for first-time low back pain Spine 1999 24 2497 505 10.1097/00007632-199912010-00012 10626313

[B23] Takala EP Viikari-Juntura E Do functional tests predict low back pain? Spine 2000 25 2126 32 10.1097/00007632-200008150-00018 10954645

[B24] Linton SJ Halldén K Can we screen for problematic back pain? A screening questionnaire for predicting outcome in acute and subacute back pain Clin J Pain 1998 14 209 15 10.1097/00002508-199809000-00007 9758070

[B25] Moon KGM Royston P Vergouwe Y Grobbee DF Altman DG Prognosis and prognostic research: what, why, and how? BMJ 2009 338 b375 10.1136/bmj.b375 19237405

[B26] Linton SJ Boersma K Early identification of patients at risk of developing a persistent back problem: the predictive validity of the Orebro Musculoskeletal Pain Questionnaire Clin J Pain 2003 19 80 6 10.1097/00002508-200303000-00002 12616177

[B27] Hill JC Dunn KM Lewis M Mullis R Main CJ Foster NE Hay EM A primary care back pain screening tool: identifying patient subgroups for initial treatment Arthritis Rheum 2008 59 632 41 10.1002/art.23563 18438893

[B28] Von Korff M Miglioretti DL A prognostic approach to defining chronic pain Pain 2005 117 304 13 10.1016/j.pain.2005.06.017 16153772

[B29] Mahatnirunkul S Tuntipivatanaskul W Pumpisanchai W Comparison of the WHOQOL-100 and the WHOQOL-BREF (26 items) J Ment Health Thai 1998 5 4 15

[B30] Mahatnirunkul S Pumpisanchai W Tapanya Pimmart. The construction of Suan Prung stress test for Thai population J Suanprung Psychiatr Hosp 2540 13 1 20

[B31] Chalernvanichakorn T Sithisarankul P Hiransuthikul N Shift work and type 2 diabetic patients' health J Med Assoc Thai 2008 91 1093 6 18839851

[B32] Buapetch A Lagampan S Faucett J Kalampakorn S The Thai version of Effort-Reward Imbalance Questionnaire (Thai ERIQ): a study of psychometric properties in garment workers J Occup Health 2008 50 480 91 10.1539/joh.L8017 18946191

[B33] Kuorinka I Jonsson B Kilbom A Vinterberg H Biering-Sørensen F Andersson G Jørgensen K Standardised Nordic questionnaires for the analysis of musculoskeletal symptoms Appl Ergon 1987 18 233 7 10.1016/0003-6870(87)90010-X 15676628

[B34] Alberti KG Zimmet P Shaw J Metabolic syndrome - a new world-wide definition. A Consensus Statement from the International Diabetes Federation Diabet Med 2006 23 469 80 10.1111/j.1464-5491.2006.01858.x 16681555

[B35] Pischon T Boeing H Hoffmann K Bergmann M Schulze MB Overvad K General and abdominal adiposity and risk of death in Europe N Engl J Med 2008 359 2105 20 10.1056/NEJMoa0801891 19005195

[B36] Petty NJ Neuromusculoskeletal examination and assessment: a handbook for therapists 2006 3 Edinburgh: Elsevier Churchill Livingstone

[B37] Halbertsma JP Goeken LN Hof AL Groothoff JW Eisma WH Extensibility and stiffness of the hamstrings in patients with nonspecific low back pain Arch Phys Med Rehabil 2001 82 232 8 10.1053/apmr.2001.19786 11239316

[B38] Magee DJ Orthopedic physical assessment 2008 5 Missouri: Saunders Elsevier

[B39] Milne JS Lauder IJ Age effects in kyphosis and lordosis in adults Ann Hum Biol 1974 1 327 37 10.1080/03014467400000351 4419577

[B40] Farasyn A Meeusen R Validity of the new Backache Index (BAI) in patients with low back pain The Spine Journal 2006 6 565 71 10.1016/j.spinee.2006.01.021 16934729

[B41] Hagins M Adler K Cash M Daugherty J Mitrani G Effects of practice on the ability to perform lumbar stabilization exercises J Orthop Sports Phys Ther 1999 29 546 55 10518297 10.2519/jospt.1999.29.9.546

[B42] Aday LA Designing & Conducting Health Surveys 1996 2 San Francisco: Jossey-Bass Publishers

[B43] Jekel FJ Elmore JG Katz DL Jekel FJ, Elmore JG, Katz DL Understanding and reducing errors in clinical medicine Epidemiology Biostatics and Preventive Medicine 1996 USA: W.B. Saunders company 85 97

[B44] WHO Expert Consultation. Appropriate body-mass index for Asian populations and its implications for policy and intervention strategies Lancet 2004 363 157 63 10.1016/S0140-6736(03)15268-3 14726171

[B45] Beach TA Parkinson RJ Stothart JP Callaghan JP Effects of prolonged sitting on the passive flexion stiffness of the in vivo lumbar spine Spine J 2005 5 145 54 10.1016/j.spinee.2004.07.036 15749614

[B46] Fritz JM Wainner RS Examining diagnostic tests: an evidence-based perspective. Examining diagnostic tests: an evidence-based perspective Phys Ther 2001 81 1546 64 11688591 10.1093/ptj/81.9.1546

[B47] Hennekens CH Buring Screening JE Mayrent SL Epidemiology in Medicine 1987 USA: Little, Brown and company 331 45

[B48] Harcombe H McBride D Derrett S Gray A Physical and psychosocial risk factors for musculoskeletal disorders in New Zealand nurses, postal workers and office workers Inj Prev 2010 16 96 100 10.1136/ip.2009.021766 20363815

